# Age and sex differences in cognitive performance in people with subjective cognitive decline and associated worry: Findings from the Canadian Longitudinal Study on Aging

**DOI:** 10.1111/jnp.70049

**Published:** 2026-04-21

**Authors:** Sofia Marinou, Vanessa Taler

**Affiliations:** ^1^ School of Psychology University of Ottawa Ontario Canada; ^2^ Bruyère Health Research Institute Ottawa Ontario Canada

**Keywords:** CLSA, cognition, executive function, memory, SCD

## Abstract

Subjective cognitive decline (SCD) refers to self‐perceived decline in cognition in the absence of objective impairment and may represent a preclinical stage of Alzheimer's disease, particularly when accompanied by worry. However, limited research has examined the influence of age and sex on cognitive performance among individuals with SCD. This study investigated age‐ and sex‐specific associations between SCD (+/− worry) and cross‐sectional cognition in the Canadian Longitudinal Study on Aging (CLSA). Participants were categorized into Controls (*n* = 5713), SCD‐No Worry (*n* = 3379) and SCD + Worry (*n* = 3511). Analyses were stratified by age (45–54, 55–64, 65–74, 75+) and sex and adjusted for education and depressive symptoms. Cognitive outcomes included four executive function and two verbal memory measures. Regarding executive function, men with SCD + Worry outperformed controls on letter fluency in the 55–64 and 65–74 groups, and women in both SCD groups outperformed controls on letter fluency in the 65–74 group. In contrast, men aged 75+ with SCD + Worry performed worse than controls on semantic fluency. No group differences emerged on Stroop Interference or the mental alternation task. For memory, men aged 45–54 and 55–64 with SCD‐No Worry had higher immediate recall, whereas in the 75+ group, lower delayed recall was observed in men with SCD (+/− worry) and women with SCD‐No Worry compared to controls. Overall, the results indicate that the clinical relevance of SCD may increase with age, independent of sex and worry status. These findings may improve the clinical utility of SCD and inform earlier detection of individuals at risk of future cognitive decline.

## INTRODUCTION

Subjective cognitive decline (SCD) is characterized by a self‐perceived decline in cognitive abilities without evidence of impairment on standardized neuropsychological tests (Jessen, Amariglio, et al., 2014). Although cognitively unimpaired by definition, individuals with SCD are at higher risk of cognitive decline relative to those without SCD, including progression to mild cognitive impairment (Koppara et al., 2015; Mitchell et al., 2014; Parfenov et al., 2020; Pike et al., 2022; van Harten et al., 2018) and dementia (Reisberg et al., 2010; Slot et al., 2019; see X.T Wang et al., 2021 for review). Moreover, SCD has been associated with the presence of biomarkers consistent with AD pathology such as elevated amyloid‐beta and tau burden (Amariglio et al., 2018; Buckley et al., 2017), as well as reduced hippocampal volumes (Perrotin et al., 2015; Scheef et al., 2012; see X. Wang et al., 2020 for review of neuroimaging studies). These findings support the growing recognition of SCD as a potential marker of preclinical AD (Jessen et al., 2020).

However, not all individuals with SCD develop cognitive impairment (see Mendonça et al., 2016 for a review), and factors such as affective symptoms (e.g., depression), personality traits (e.g., neuroticism) and medication use can contribute to SCD (Jessen, Amariglio, et al., 2014). As a result, identifying individuals whose subjective concern signals emerging pathological decline remains a challenge. To address this, the SCD‐*plus* framework (Jessen, Amariglio, et al., 2014) was introduced to identify features of SCD that are associated with a higher likelihood of underlying AD pathology. One such feature is SCD‐related worry, which refers to a persistent concern about perceived cognitive decline. Individuals with SCD‐related worry have higher rates of AD biomarker positivity (Kuhn et al., 2025), as well as a greater likelihood of cognitive decline (Pike et al., 2022; Snitz et al., 2018) and progression to dementia (Jessen et al., 2010; Jessen, Wolfsgruber, et al., 2014; Wolfsgruber et al., 2016), compared to those without worry.

Although some longitudinal studies suggest that SCD is associated with an increased risk of poorer cognitive outcomes over time (see Rabin et al., 2017; Zhou et al., 2025 for reviews), cross‐sectional findings are mixed. Several studies report lower performance across various cognitive domains in individuals with SCD (e.g., Benito‐León et al., 2010; Kim et al., 2020; López‐Higes et al., 2025; Morrison & Oliver, 2022; Park et al., 2019; Wolfsgruber et al., 2020), with deficits in episodic memory emerging most often (Zhou et al., 2025). Others, however, find no observable differences compared to individuals without SCD (see Burmester et al., 2016 for review). These inconsistencies may be partly due to the limited examination of important demographic moderators, such as age and sex, which may influence the clinical relevance of SCD. Given the substantial heterogeneity among individuals with SCD, assessing these moderating factors is crucial for understanding who is most at risk.

Age is the biggest risk factor for dementia. However, distinguishing between typical age‐related changes and early neurodegeneration can be challenging, especially in SCD where subjective concerns may emerge years before a dementia diagnosis (Gauthier et al., 2006; Jessen et al., 2020; Verlinden et al., 2016). Previous research has suggested that there may be an “optimal” age window during which SCD may be most informative for identifying AD risk (Molinuevo et al., 2017; L. Wang et al., 2004). For example, SCD is more likely to be associated with non‐AD factors, such as depression, in individuals under 60 (Jessen, Amariglio, et al., 2014). Similarly, in individuals in their 80s, cognitive complaints are more common and less specific, thereby reducing their predictive value for dementia (L. Wang et al., 2004). Despite this, few studies have examined whether the association between SCD and cognitive performance differs across midlife and older adulthood.

In addition to age, sex is also important to consider when assessing cognition in individuals with SCD. Higher prevalence of SCD has been found in women relative to men (e.g., Cheng et al., 2023; Kim et al., 2020; Tomita et al., 2014), although this varies across studies (see Holmen et al., 2013; Röhr et al., 2020; Schliep et al., 2022; L. Wang et al., 2004). Women with SCD are also more likely to report SCD‐related worry (Heser et al., 2019), experience more cognitive decline (Oliver et al., 2022) and progress to dementia at higher rates than men with SCD (Heser et al., 2019; Pérès et al., 2011). Given that women are disproportionally affected by AD (Association et al., 2013; Gao et al., 1998; Mielke, 2018), these patterns raise the possibility that SCD may have different clinical implications for women than for men. However, few studies have explicitly examined the role of sex in the relationship between SCD and cognition.

Taken together, the existing research supports the relevance of SCD as a potential indicator of preclinical AD. However, cross‐sectional findings remain inconsistent, with studies often failing to examine potential moderating factors such as age and sex. To address these gaps, this study uses data from the Canadian Longitudinal Study on Aging (CLSA) to perform age‐ and sex‐stratified analyses examining differences in cognitive performance between older adults with and without SCD (+/− worry). Specifically, we focus on memory and executive function. By identifying when and in whom cross‐sectional cognitive differences emerge, this study aims to improve the clinical utility of SCD and inform earlier detection of individuals at risk of future cognitive decline.

## METHODS

Data were taken from the first three‐year follow‐up (FU1) of the CLSA, a 20‐year national study following approximately 50,000 Canadians aged 45–85 at baseline (Raina et al., 2009). SCD was first assessed at FU1, and as such, only data from this wave were used in the current analyses. The CLSA consists of two cohorts: the Tracking cohort (*n* = 21,241), who complete phone interviews, and the Comprehensive cohort (*n* = 30,097), who complete in‐person interviews. This study only includes data from the Comprehensive cohort because some cognitive measures were only collected within this group.

CLSA eligibility criteria included being able to speak English or French. Individuals were excluded at baseline if they had a diagnosis of cognitive impairment, lived in Canadian territories or other remote regions, lived in First Nations reserves or First Nations settlements, were a full‐time member of the Canadian Armed Forces or were institutionalized (see Raina et al., 2009, 2019 for detailed information on the CLSA sampling frame). For the present analyses, additional exclusion criteria were applied at FU1. Participants were excluded if they did not complete neuropsychological testing in person (*n* = 1790) or in English (*n* = 5004), did not respond to SCD‐related questions (*n* = 167), or self‐reported a formal diagnosis of dementia, a memory problem, a neurological condition, or traumatic brain injury with loss of consciousness (*n* = 3876). Participants were also excluded if they did not complete two or more of the six cognitive measures assessed (*n* = 2157) or met criteria for cognitive impairment (*n* = 747; see below). Ethics approval for this secondary analysis was acquired from the Bruyère Health Research Institute and the University of Ottawa ethics boards. Informed consent was provided by all participants.

### Measures

Information pertaining to sex, age, education and depressive symptoms was self‐reported by participants through in‐person structured interviews. The level of education was measured by asking participants to report the highest level of education they have received, with responses falling into four categories: < high school, high school, some post‐secondary and post‐secondary. Depressive symptoms were assessed using the Center for Epidemiologic Studies Short Depression Scale (CES‐D10; Andresen et al., 1994), which evaluates the frequency of depressive symptoms experienced in the past week. Total scores range from 0 to 30, with scores higher than 10 indicating depression.

#### Subjective cognitive decline

SCD was measured at FU1 by asking participants the question, “Do you feel like your memory is becoming worse?” Participants who responded “no” were classified as non‐SCD controls. Those who responded “yes” were subsequently asked, “Does this worry you?” This follow‐up question gave an indication of SCD‐related worry on a 5‐point Likert scale ranging from strongly agree to strongly disagree. Participants who endorsed strongly agree or agree were placed in the SCD + Worry group, and those who responded with disagree or strongly disagree were placed in the SCD‐No Worry group. Those who responded with undecided (*n* = 1197) were excluded, as SCD‐related worry could not be determined.

To ensure that all included participants were cognitively unimpaired at FU1, cognitive impairment was defined as scoring more than 1.5 SD below the control group mean on at least two tests within the same cognitive domain (Jessen, Amariglio, et al., 2014). Participants meeting this criterion (*n* = 747) were excluded.

### Executive function tasks

#### Controlled oral word association test (COWAT)

The COWAT (Lezak et al., 2004) is a measure of letter fluency. Three 60‐s trials are administered, in which participants are instructed to generate as many words as possible beginning with a target letter (i.e., F, A, and S). One point is awarded for each acceptable word. Unacceptable words include proper nouns and words with the same root but different endings (e.g., eat, eating). The total COWAT score is calculated by summing scores across all three trials, with higher scores indicating better performance.

#### Animal fluency task (AFT)

The AFT (Read, 1987) evaluates semantic fluency. Participants are asked to name as many animals as possible in 60 s. In the CLSA, performance is scored using two algorithms: a strict algorithm, where points are awarded only for taxonomically distinct species‐level responses (e.g., fish and dog), and a lenient algorithm that assigns points for each unique animal named. This present study uses scores derived from the lenient algorithm. Higher scores indicate better performance.

#### Mental alternation test (MAT)

The MAT (Teng, 1995) measures cognitive flexibility, processing speed and task switching. It consists of two parts: in part A, participants are instructed to count aloud from 1 to 20 and then recite the alphabet. In part B, participants must alternate between the numbers 1 and 26 and the alphabet, beginning with the letter A (i.e., 1‐A, 2‐B, 3‐C, 4‐D, etc.) for 30 s. The final score is the number of correct alternations, discounting errors, with scores ranging from 0 to 51. Higher scores indicate better performance.

#### Stroop neurological screening test (Victoria version)

The Stroop test (Victoria Version; Bayard et al., 2011; Troyer et al., 2006) is a measure of executive function assessing inhibition, attention and mental speed and control. The task consists of three parts: (1) the Dot trial, in which participants are asked to name the colour of solid‐coloured circles; (2) the Word trial, in which they name the ink colour of non‐colour words; and (3) the Colour‐Word trial, where they name the ink colour of incongruent colour words (e.g., the word “blue” printed in red ink). To account for the influence of age‐related changes in processing speed, Stroop interference scores are calculated by dividing the time to complete the Colour‐Word trial by the time of the Dot Trial (Strauss et al., 2006). A shorter interference time indicates better performance.

### Memory tasks

#### Rey auditory verbal learning test (RAVLT)

A modified version of the RAVLT (Rey, 1964) was administered to assess verbal learning and memory. Participants listened to a recording of a list of fifteen unrelated nouns read at a rate of one word per second, followed by one immediate free recall trial. After a five‐minute delay, delayed recall was assessed in a single trial. Each trial is scored by the total number of correctly recalled words for a maximum of fifteen points per trial. A higher score indicates better performance.

### Statistical analyses

Statistical analyses were performed using R Statistical Software (v4.4.2; R Core Team 2024). Univariate and multivariate outliers (*n* = 506) were identified and removed based on the procedures outlined by Raykov and Marcoulides (2008). Univariate outliers were identified using Q–Q plots, boxplots and z‐scores, with values exceeding ±3 SD classified as outliers. Multivariate outliers were identified using Mahalanobis distance: cases exceeding the chi‐square critical value at α = .001 (based on the relevant degrees of freedom) were excluded. The distribution of continuous variables was normal as evaluated using Q–Q plots and skew statistics (−2< and >2; Curran et al., 1996). The proportion of missing data for neuropsychological test variables was low (<5% per variable). Given the minimal extent of missingness, we applied mean imputation at the variable level. As such, all analyses were conducted using complete case data.

Group differences on continuous demographic variables were analysed using univariate analysis of variance (ANOVA), contrasts corrected with the Holm‐Bonferroni procedure. Categorical data were analysed using chi‐square and Kruskal–Wallis tests, and pairwise comparisons were conducted using pairwise Wilcoxon tests with Holm–Bonferroni corrections. Differences in cognitive performance between the control group and the two SCD subgroups were examined using multiple linear regression models, stratified by both age (45–54, 55–64, 65–74, 75+) and sex (male, female). Education level and depression scores were included in the models as covariates. All continuous predictors were mean‐centered prior to analysis. To correct for multiple comparisons, *p*‐values were adjusted using the Benjamini–Hochberg procedure. For all analyses, statistical significance was established at *p* < .05.

## RESULTS

Demographic characteristics of the sample are displayed in Table 1. After exclusion data were applied, participants were categorized into three groups, resulting in a final sample of *n* = 5713 in the Control group, *n* = 3379 in the SCD‐No Worry group and *n* = 3511 in the SCD + Worry group. Significant group differences were found in age (*F* (2, 12,600) = 30.40, *p* < .001, *η* ^*2*^ = .005), with the SCD‐No Worry group being slightly older than both the Controls (*MD* = −.67, *SE =* .09, 95% CI [−.83, −.50]) and the SCD + Worry group (*MD* = −1.57, *SE =* .09, 95% CI [−1.76, −1.39]).

**TABLE 1:** Participant demographic characteristics.

	Controls (*n* = 5713)	SCD‐No Worry (*n* = 3379)	SCD + Worry (*n* = 3511)
Age, mean (SD)*	64.1 (9.4)	65.7 (10.2)	64.3 (9.5)
Age group, *n* (%)			
45–54	1003 (17.6)	540 (16.0)	608 (17.3)
55–64	2112 (37.0)	1088 (32.2)	1299 (37.0)
65–74	1697 (29.7)	1020 (30.2)	1011 (28.8)
75+	901 (15.8)	731 (21.6)	593 (16.9)
Sex, *n* (%)*			
Male	2888 (50.6)	1731 (52.1)	1467 (41.8)
Female	2825 (49.4)	1648 (47.8)	2044 (58.2)
Education, *n* (%)			
< High school	151 (2.6)	96 (2.8)	70 (2.0)
High school	433 (7.6)	275 (8.1)	254 (7.2)
Some post‐secondary	463 (8.1)	252 (7.5)	307 (8.7)
Post‐secondary	4666 (81.7)	2756 (81.6)	2880 (82.0)
Depression, mean (SD)*/30	3.8 (3.6)	4.4 (3.8)	6.0 (4.6)

Abbreviation: SCD = Subjective Cognitive Decline.

* Indicates significant group differences at *p* < .001.

Significant group differences were observed for sex (*χ* ^*2*^ (2) = 82.91, *p* < .001), with a higher proportion of female participants in the SCD + Worry group relative to both the control group and the SCD‐No Worry group (all *ps* < .001). Depression levels differed significantly across groups (*F* (2, 12,600) = 353.53, *p* < .001, *η* ^*2*^ = .05): participants in the control group reported fewer depressive symptoms than the SCD‐No Worry group (*MD* = −.67, *SE* = .09, 95% CI [−.83, −.50]) and the SCD + Worry group (*MD* = −2.24, *SE* = .08, 95% CI [−2.40, −2.07]). Moreover, the SCD + Worry group had more depressive symptoms than the SCD‐No Worry group (*MD =* 1.57, *SE =* .09, 95% CI [−1.76, −1.39]). No significant differences were found for educational attainment (*χ* ^*2*^ (2) = .75, *p =* .689) between groups.

### Executive function

**FIGURE 1:**
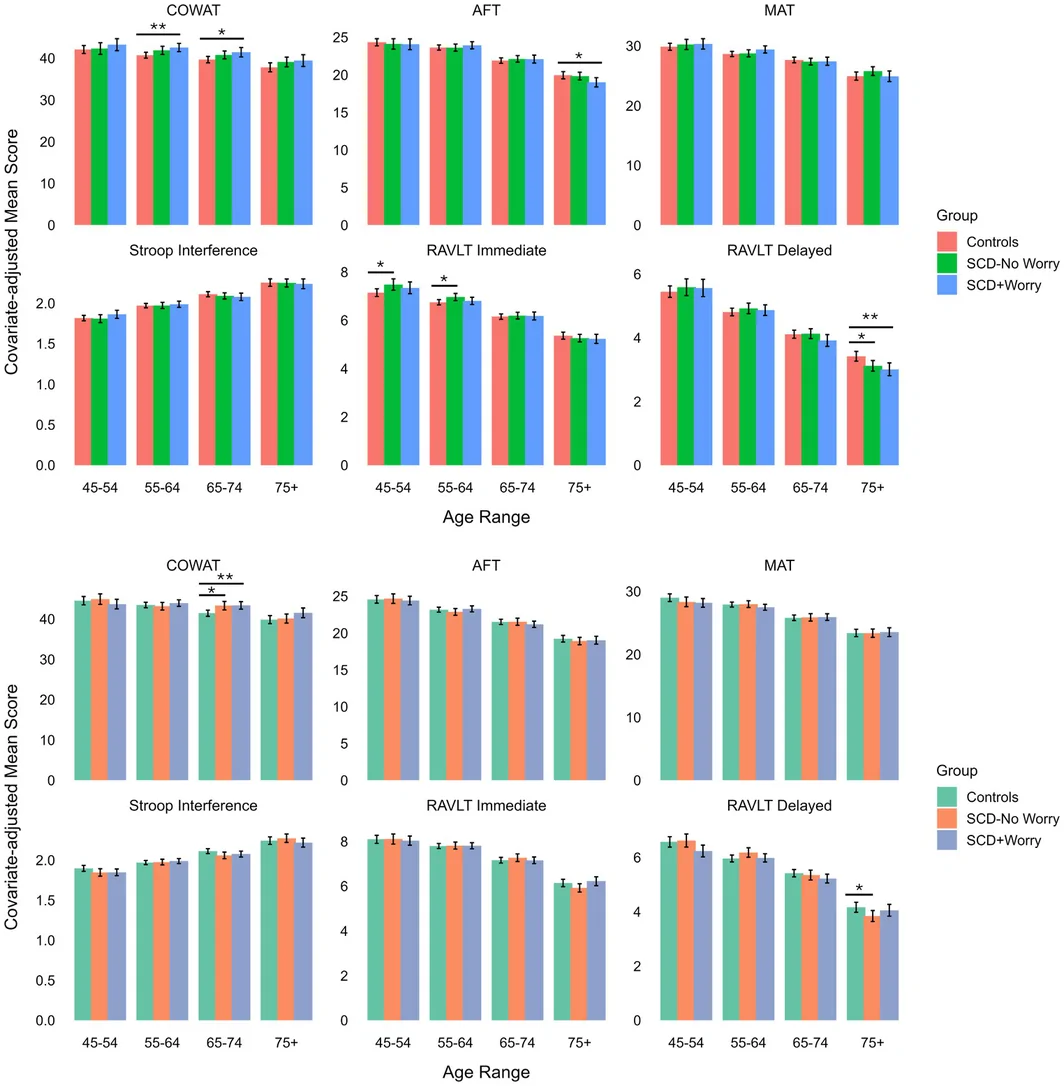
Mean neuropsychological test scores of study samples for men (top panel) and women (bottom panel), stratified by age. Scores reflect estimated marginal means, adjusted for education level and depression scores. Bars indicate 95% CIs. SCD = Subjective Cognitive Decline; COWAT = Controlled Oral Word Association Test; AFT = Animal Fluency Test; MAT = Mental Alternation Test; RAVLT = Rey Auditory Verbal Learning Test. * *p* < .05; ** *p* < .01.

Complete results from age‐ and sex‐stratified linear regression models examining associations between SCD status and executive function performance are presented in Tables 2 and 3 for men and women, respectively, and illustrated in Figure 1.

**TABLE 2:** Linear regression results for executive function measures by age in men.

	COWAT					AFT					MAT					Stroop Interference				
	*b* [95% CI]	SE	*β* [95% CI]	*t*	*p*	*b* [95% CI]	SE	*β* [95% CI]	*t*	*p*	*b* [95% CI]	SE	*β* [95% CI]	*t*	*p*	*b* [95% CI]	SE	*β* [95% CI]	*t*	*p*
45–54																				
SCD‐No Worry	.24 [−1.44, 1.93]	.86	.02 [−.13, .17]	.28	.833	−.23 [−1.07, .62]	.43	−.04 [−.19, .11]	−.53	.678	.38 [−.65, 1.41]	.53	.05 [−.09, .20]	.73	.571	−.01 [−.07, .05]	.03	−.01 [−.14, .11]	−.23	.816
SCD + Worry	1.17 [−.56, 2.90]	.88	.10 [−.05, .25]	1.33	.267	−.27 [−1.13, .60]	.44	−.05 [−.20, .11]	−.60	.650	.48 [−.58, 1.53]	.54	.07 [−.08, .22]	.89	.492	.05 [−.01, .11]	.03	.10 [−.03, .23]	1.50	.133
Education	2.93 [1.61, 4.24]	.67	.18 [.10, .27]	4.37	< .001	.65 [−.01, 1.31]	.34	.08 [−.00, .17]	1.95	.093	1.09 [.29, 1.89]	.41	.11 [.03, .19]	2.67	.017	−.04 [−.08, .01]	.02	−.06 [−.13, .01]	−1.66	.152
Depression	−.16 [−.35, .03]	.10	−.06 [−.12, .01]	−1.66	.152	−.15 [−.24, −.05]	.05	−.10 [−.17, −.04]	−3.05	.006	−.16 [−.27, −.04]	.06	−.09 [−.16, −.02]	−2.68	.017	.01 [−.00, .01]	.00	.05 [−.01, .11]	1.71	.143
55–64																				
SCD‐No Worry	1.15 [−.06, 2.36]	.62	.10 [−.01, .21]	1.86	.109	−.01 [−.59, .58]	.30	−.00 [−.10, .10]	−.03	.974	.11 [−.62, .84]	.37	.02 [−.09, .12]	.29	.833	.00 [−.04, .05]	.02	.01 [−.09, .11]	.11	.927
SCD + Worry	1.83 [.59, 3.07]	.63	.16 [.05, .27]	2.90	.009	.30 [−.29, .90]	.31	.05 [−.05, .16]	1.00	.435	.76 [.02, 1.51]	.38	.11 [.00, .22]	2.00	.082	.02 [−.03, .06]	.02	.04 [−.07, .14]	.69	.590
Education	1.84 [1.07, 2.60]	.39	.11 [.07, .16]	4.71	< .001	.82 [.45, 1.19]	.19	.10 [.06, .15]	4.37	< .001	.82 [.36, 1.29]	.24	.08 [.04, .13]	3.50	.001	−.07 [−.10, −.04]	.02	−.11 [−.15, −.06]	−4.59	< .001
Depression	−.08 [−.22, .05]	.07	−.03 [−.08, .02]	−1.21	.322	−.08 [−.15, −.02]	.03	−.06 [−.11, −.01]	−2.45	.030	−.18 [−.26, −.10]	.04	−.11 [−.15, −.06]	−4.37	< .001	−.00 [−.01, .00]	.00	−.00 [−.05, .04]	−.19	.887
65–74																				
SCD‐No Worry	1.12 [−.10, 2.33]	.62	.10 [−.01, .20]	1.80	.120	.23 [−.34, .80]	.29	.04 [−.06, .14]	.79	.552	−.27 [−1.01, .46]	.38	−.04 [−.14, .07]	−.73	.571	−.02 [−.07, .03]	.03	−.04 [−.15, .06]	−.79	.552
SCD + Worry	1.76 [.39, 3.14]	.70	.15 [.03, .27]	2.51	.026	.19 [−.45, .84]	.33	.03 [−.08, .15]	.59	.650	−.22 [−1.06, .62]	.43	−.03 [−.15, .09]	−.51	.681	−.03 [−.09, .02]	.03	−.07 [−.19, .05]	−1.11	.374
Education	2.03 [1.28, 2.77]	.38	.13 [.08, .17]	5.34	< .001	1.00 [.65, 1.35]	.18	.13 [.08, .17]	5.59	< .001	1.27 [.82, 1.73]	.23	.13 [.08, .18]	5.52	< .001	−.05 [−.08, −.02]	.02	−.08 [−.13, −.03]	−3.32	.002
Depression	−.15 [−.30, −.00]	.08	−.05 [−.11, −.00]	−2.02	.080	−.12 [−.19, −.05]	.04	−.08 [−.13, −.03]	−3.28	.003	−.21 [−.30, −.12]	.05	−.12 [−.17, −.07]	−4.55	< .001	.00 [−.00, .01]	.00	.02 [−.03, .07]	.76	.562
75+																				
SCD‐No Worry	1.29 [−.30, 2.88]	.81	.11 [−.03, .25]	1.59	.170	−.11 [−.81, .58]	.35	−.02 [−.14, .10]	−.32	.814	.83 [−.16, 1.82]	.50	.12 [−.02, .26]	1.65	.154	−.00 [−.07, .06]	.03	−.01 [−.16, .13]	−.14	.909
SCD + Worry	1.62 [−.18, 3.42]	.92	.14 [−.02, .30]	1.76	.128	−.93 [−1.72, −.15]	.40	−.16 [−.30, −.03]	−2.33	.041	−.03 [−1.16, 1.09]	.57	−.00 [−.17, .16]	−.06	.960	−.01 [−.09, .06]	.04	−.03 [−.20, .13]	−.38	.779
Education	2.60 [1.81, 3.40]	.40	.16 [.11, .21]	6.45	< .001	.72 [.38, 1.07]	.18	.09 [.05, .14]	4.10	< .001	1.41 [.92, 1.91]	.25	.15 [.09, .20]	5.62	< .001	−.04 [−.07, −.00]	.02	−.06 [−.11, −.00]	−2.08	.07
Depression	−.06 [−.26, .14]	.10	−.02 [−.09, .05]	−.57	.654	.03 [−.05, .12]	.04	.02 [−.04, .09]	.75	.569	−.11 [−.24, .01]	.06	−.07 [−.14, .01]	−1.76	.128	.01 [.00, .02]	.00	.08 [.01, .16]	2.19	.055

*Note*: Table reports both unstandardized (b) and standardized (β) regression coefficients with 95% confidence intervals; SEs are unstandardized; *p* values are Benjamini–Hochberg corrected.

Abbreviations: SCD = Subjective Cognitive Decline; COWAT = Controlled Oral Word Association Test; AFT = Animal Fluency Test; MAT = Mental Alternation Test.

**TABLE 3:** Linear regression results for executive function measures by age in women.

	COWAT					AFT					MAT					Stroop Interference				
	*b* [95% CI]	SE	*β* [95% CI]	*t*	*p*	*b* [95% CI]	SE	*β* [95% CI]	*t*	*p*	*b* [95% CI]	SE	*β* [95% CI]	*t*	*p*	*b* [95% CI]	SE	*β* [95% CI]	*t*	*p*
45–54																				
SCD‐No Worry	.39 [−1.27, 2.05]	.85	.03 [−.11, .18]	.46	.773	.10 [−.72, .92]	.42	.02 [−.13, .16]	.24	.894	−.66 [−1.64, .31]	.50	−.10 [−.23, .04]	−1.34	.276	−.05 [−.11, .01]	.03	−.11 [−.24, .02]	−1.68	.147
SCD + Worry	−.84 [−2.43, .75]	.81	−.07 [−.21, .07]	−1.04	.429	−.16 [−.95, .63]	.40	−.03 [−.17, .11]	−.40	.802	−.82 [−1.75, .12]	.48	−.12 [−.25, .02]	−1.72	.137	−.05 [−.11, .01]	.03	−.11 [−.23, .02]	−1.72	.137
Education	2.03 [.85, 3.21]	.60	.10 [.04, .16]	3.38	.002	1.10 [.52, 1.69]	.30	.1 [.07, .21]	3.70	< .001	1.05 [.36, 1.75]	.35	.11 [.04, .18]	2.99	.007	−.05 [−.09, −.01]	.02	−.08 [−.14, −.01]	−2.35	.039
Depression	−.08 [−.24, .08]	.08	−.03 [−.09, .03]	−.95	.478	−.03 [−.11, .06]	.04	−.02 [−.08, .04]	−.62	.675	−.12 [−.22, −.03]	.05	−.07 [−.13, −.02]	−2.54	.026	−.00 [−.00, .01]	.00	.03 [−.02, .08]	1.24	.323
55–64																				
SCD‐No Worry	−.31 [−1.45, .84]	.58	−.03 [−.13, .07]	−.52	.728	−.30 [−.86, .26]	.29	−.05 [−.15, .05]	−1.05	.429	.07 [−.59, .72]	.34	.01 [−.08, .10]	.20	.905	.01 [−.04, .05]	.02	.01 [−.08, .11]	.25	.891
SCD + Worry	.46 [−.60, 1.53]	.54	.04 [−.05, .13]	.85	.530	.11 [−.42, .63]	.27	.02 [−.07, .11]	.40	.802	−.43 [−1.04, .18]	.31	−.06 [−.15, .03]	−1.38	.259	.02 [−.02, .06]	.02	.04 [−.05, .13]	.85	.530
Education	1.70 [1.03, 2.37]	.34	.11 [.06, .15]	4.98	< .001	.90 [.57, 1.23]	.17	.11 [.07, .16]	5.37	< .001	.41 [.03, .80]	.20	.04 [.00, .08]	2.11	.066	−.05 [−.08, −.03]	.01	−.08 [−.12, −.04]	−4.06	< .001
Depression	−.12 [−.23, −.02]	.05	−.04 [−.08, −.01]	−2.30	.044	−.06 [−.11, −.01]	.03	−.04 [−.08, −.01]	−2.37	.039	−.13 [−.18, −.07]	.03	−.07 [−.11, −.04]	−4.10	< .001	.01 [.00, .01]	.00	.05 [.01, .08]	2.70	.016
65–74																				
SCD‐No Worry	1.87 [.58, 3.15]	.66	.16 [.05, .27]	2.84	.011	.02 [−.58, .62]	.31	.00 [−.10, .11]	.08	.963	.06 [−.67, .80]	.37	.01 [−.10, .11]	.17	.913	−.05 [−.10, −.00]	.03	−.11 [−.22, −.00]	−2.00	.080
SCD + Worry	1.93 [.72, 3.14]	.62	.17 [.06, .27]	3.13	.005	−.33 [−.89, .23]	.29	−.06 [−.16, .04]	−1.16	.367	.12 [−.57, .80]	.35	.02 [−.08, .12]	.33	.843	−.04 [−.08, .01]	.02	−.08 [−.18, .03]	−1.46	.225
Education	1.67 [1.01, 2.33]	.34	.10 [.06, .15]	4.95	< .001	.90 [.59, 1.20]	.16	.11 [.07, .15]	5.72	< .001	.35 [−.03, .73]	.19	.04 [−.00, .07]	1.82	.117	−.03 [−.06, −.01]	.01	−.05 [−.09, −.01]	−2.46	.031
Depression	−.18 [−.30, −.06]	.06	−.06 [−.10, −.02]	−2.92	.009	−.06 [−.12, −.00]	.03	−.04 [−.08, −.00]	−2.14	.063	−.13 [−.19, −.06]	.03	−.07 [−.11, −.03]	−3.66	< .001	.00 [.00, .01]	.00	.04 [.00, .08]	2.00	.080
75+																				
SCD‐No Worry	.27 [−1.22, 1.77]	.76	.02 [−.11, .15]	.36	.830	−.32 [−.99, .35]	.34	−.06 [−.17, .06]	−.93	.487	−.03 [−.91, .85]	.45	−.00 [−.13, .12]	−.07	.963	.03 [−.04, .10]	.04	.06 [−.09, .22]	.85	.530
SCD + Worry	1.68 [.11, 3.25]	.80	.15 [.01, .28]	2.09	.067	−.20 [−.90, .51]	.36	−.03 [−.16, .09]	−.55	.713	.13 [−.80, 1.05]	.47	.02 [−.11, .15]	.27	.887	−.02 [−.10, .05]	.04	−.05 [−.21, .11]	−.65	.657
Education	1.83 [1.15, 2.50]	.35	.11 [.07, .16]	5.29	< .001	.68 [.38, .99]	.15	.09 [.05, .13]	4.41	< .001	.78 [.38, 1.18]	.20	.08 [.04, .12]	3.82	< .001	−.04 [−.07, −.01]	.02	−.06 [−.11, −.01]	−2.45	.032
Depression	−.14 [−.29, .02]	.08	−.05 [−.10, .01]	−1.77	.130	−.07 [−.14, .00]	.04	−.05 [−.10, .00]	−1.88	.106	−.15 [−.24, −.06]	.05	−.09 [−.14, −.03]	−3.18	.004	.00 [−.00, .01]	.00	.02 [−.04, .08]	.70	.626

*Note*: Table reports both unstandardized (b) and standardized (β) regression coefficients with 95% confidence intervals; SEs are unstandardized; *p* values are Benjamini–Hochberg corrected.

Abbreviations: SCD = Subjective Cognitive Decline; COWAT = Controlled Oral Word Association Test; AFT = Animal Fluency Test; MAT = Mental Alternation Test.

Among men, individuals aged 55–64 and 65–74, the SCD + Worry group outperformed the control group on the COWAT (55–64: *b* = 1.83, 95% CI [.59, 3.07], *p* = .009; 65–74: *b* = 1.76, 95% CI [.39, 3.14], *p* = .026). However, in the 75+ age group, the SCD + Worry group had worse performance than controls on the AFT (*b* = −.93, 95% CI [−1.72, −.15], *p* = .041).

Among women, in the 65–74 age group, both SCD groups outperformed controls on the COWAT (SCD‐No Worry: *b* = 1.87, 95% CI [.58, 3.15], *p* = .011; SCD‐Worry: *b* = 1.93, 95% CI [.72, 3.14], *p* = .005). No significant group differences were observed in the youngest age group (45–54) on any executive function task for either men or women.

### Memory

Complete results from age‐ and sex‐stratified linear regression models examining associations between SCD status and memory performance are presented in Tables 4 and 5 for men and women, respectively, and illustrated in Figure 1.

**TABLE 4:** Linear regression results for memory measures by age in men.

	RAVLT Immediate					RAVLT Delayed				
	*b* [95% CI]	SE	*β* [95% CI]	*t*	*p*	*b* [95% CI]	SE	*β* [95% CI]	*t*	*p*
45–54										
SCD‐No Worry	.34 [.05, .62]	.15	.17 [.03, .31]	2.30	.042	.14 [−.18, .46]	.16	.06 [−.08, .21]	.86	.512
SCD + Worry	.20 [−.09, .49]	.15	.10 [−.05, .25]	1.33	.267	.12 [−.21, .44]	.17	.05 [−.10, .20]	.69	.590
Education	.31 [.09, .53]	.11	.11 [.03, .19]	2.72	.015	.46 [.21, .71]	.13	.15 [.07, .23]	3.59	< .001
Depression	−.04 [−.07, −.01]	.02	−.08 [−.15, −.02]	−2.43	.031	−.03 [−.06, .01]	.02	−.05 [−.11, .02]	−1.40	.238
55–64										
SCD‐No Worry	.22 [.03, .40]	.09	.11 [.02, .20]	2.32	.041	.11 [−.09, .32]	.10	.05 [−.04, .14]	1.07	.391
SCD + Worry	.06 [−.13, .24]	.10	.03 [−.07, .12]	.59	.650	.06 [−.15, .27]	.11	.03 [−.07, .12]	.55	.664
Education	.37 [.25, .48]	.06	.13 [.09, .17]	6.24	< .001	.42 [.29, .55]	.07	.14 [.09, .18]	6.34	< .001
Depression	−.03 [−.05, −.01]	.01	−.07 [−.11, −.03]	−3.14	.004	−.04 [−.06, −.02]	.01	−.08 [−.12, −.03]	−3.47	.001
65–74										
SCD‐No Worry	.04 [−.14, .22]	.09	.02 [−.07, .11]	.46	.718	.02 [−.18, .22]	.10	.01 [−.08, .10]	.18	.889
SCD + Worry	.03 [−.18, .23]	.10	.01 [−.09, .12]	.25	.850	−.20 [−.42, .03]	.12	−.09 [−.19, .01]	−1.69	.145
Education	.33 [.22, .44]	.06	.12 [.08, .16]	5.87	< .001	.32 [.20, .44]	.06	.10 [.06, .14]	5.13	< .001
Depression	−.02 [−.04, −.00]	.01	−.05 [−.09, −.00]	−2.06	.075	−.02 [−.05, .00]	.01	−.04 [−.09, .00]	−1.87	.109
75+										
SCD‐No Worry	−.10 [−.32, .11]	.11	−.05 [−.16, .06]	−.94	.462	−.30 [−.52, −.07]	.11	−.13 [−.24, −.03]	−2.58	.022
SCD + Worry	−.13 [−.38, .11]	.12	−.07 [−.19, .05]	−1.09	.386	−.41 [−.66, −.15]	.13	−.18 [−.30, −.07]	−3.13	.004
Education	.22 [.11, .33]	.05	.08 [.04, .12]	4.01	< .001	.20 [.09, .31]	.06	.06 [.03, .10]	3.45	.002
Depression	−.01 [−.04, .01]	.01	−.03 [−.08, .03]	−.95	.459	−.03 [−.06, .00]	.01	−.05 [−.10, .00]	−1.84	.113

*Note*: Table reports both unstandardized (b) and standardized (β) regression coefficients with 95% confidence intervals; SEs are unstandardized; *p* values are Benjamini–Hochberg corrected.

Abbreviations: SCD = Subjective Cognitive Decline; RAVLT = Rey Auditory Verbal Learning Test.

**TABLE 5:** Linear regression results for memory measures by age in women.

	RAVLT Immediate					RAVLT Delayed				
	*b* [95% CI]	SE	*β* [95% CI]	*t*	*p*	*b* [95% CI]	SE	*β* [95% CI]	*t*	*p*
45–54										
SCD‐No Worry	.01 [−.28, .30]	.15	.01 [−.14, .15]	.08	.963	.05 [−.26, .36]	.16	.02 [−.12, .17]	.33	.843
SCD + Worry	−.06 [−.34, .22]	.14	−.03 [−.17, .11]	−.42	.800	−.34 [−.63, −.04]	.15	−.16 [−.30, −.02]	−2.25	.050
Education	.39 [.19, .60]	.10	.14 [.06, .22]	3.75	< .001	.29 [.08, .51]	.11	.10 [.02, .14]	2.64	.019
Depression	.00 [−.03, .03]	.01	.01 [−.05, .07]	.17	.913	−.00 [−.03, .03]	.02	−.00 [−.06, .06]	−.00	.998
55–64										
SCD‐No Worry	.02 [−.17, .21]	.10	.01 [−.09, .11]	.20	.905	.22 [.01, .44]	.11	.11 [.00, .21]	2.02	.078
SCD + Worry	.01 [−.17, .19]	.09	.01 [−.09, .10]	.11	.953	.02 [−.18, .22]	.10	.01 [−.08, .11]	.22	.903
Education	.25 [.14, .36]	.06	.09 [.05, .13]	4.31	< .001	.25 [.13, .38]	.06	.08 [.04, .12]	3.94	< .001
Depression	−.03 [−.05, −.01]	.01	−.07 [−.11, −.03]	−3.32	.003	−.03 [−.05, −.01]	.01	−.07 [−.11, −.03]	−3.46	.002
65–74										
SCD‐No Worry	.11 [−.10, .32]	.11	.06 [−.05, .17]	1.04	.429	−.07 [−.29, .16]	.12	−.03 [−.15, .08]	−.58	.695
SCD + Worry	−.00 [−.20, .20]	.10	−.00 [−.11, .11]	−.03	.985	−.20 [−.41, .02]	.11	−.10 [−.21, .01]	−1.81	.118
Education	.20 [.09, .31]	.06	.08 [.04, .13]	3.64	< .001	.27 [.15, .39]	.06	.10 [.06, .15]	4.55	< .001
Depression	−.04 [−.06, −.02]	.01	−.10 [−.15, −.05]	−4.29	< .001	−.03 [−.05, −.01]	.01	−.07 [−.12, −.02]	−2.99	.007
75+										
SCD‐No Worry	−.22 [−.47, .03]	.13	−.12 [−.26, .02]	−1.74	.136	−.32 [−.59, −.05]	.14	−.16 [−.30, −.02]	−2.31	.044
SCD + Worry	.08 [−.18, .34]	.13	.04 [−.10, .19]	.60	.684	−.11 [−.39, .18]	.15	−.06 [−.20, .09]	−.74	.597
Education	.13 [.01, .24]	.06	.07 [.01, .12]	2.19	.056	.14 [.01, .26]	.06	−.03 [−.09, .03]	2.19	.056
Depression	−.01 [−.04, .01]	.01	−.03 [−.09, .03]	−.82	.544	−.01 [−.04, .01]	.01	−.03 [−.09, .03]	−.95	.478

*Note*: Table reports both unstandardized (b) and standardized (β) regression coefficients with 95% confidence intervals; SEs are unstandardized; *p* values are Benjamini–Hochberg corrected.

Abbreviations: SCD = Subjective Cognitive Decline; RAVLT = Rey Auditory Verbal Learning Test.

Among men, the SCD‐No Worry group outperformed the controls on the immediate recall of the RAVLT in the 45–54 (*b* = .34, 95% CI [.05, .62], *p* = .042) and 55–64 (*b* = .22, 95% CI [.03, .40], *p* = .041) age ranges. In contrast, in the 75+ age range, both SCD groups had worse delayed recall on the RAVLT relative to the control group (SCD‐No Worry: *b* = −.30, 95% CI [−.52, −.07], *p* = .022; SCD + Worry: *b* = −.41, 95% CI [−.66, −.15], *p* = .004). Among women, the SCD‐No Worry group had lower delayed recall RAVLT scores relative to controls (*b* = −.32, 95% CI [−.59, −.05], *p* = .044) in the 75+ age range.

## DISCUSSION

The present study used data from the CLSA to examine cross‐sectional associations between SCD (+/− worry) and objective cognitive performance across age and sex groups. By stratifying our analyses by age (45–54, 55–64, 65–74, 75+) and by sex, we aimed to investigate how SCD relates to cognitive function across multiple cognitive domains and demographic subgroups. Overall, our findings suggest that SCD may be more clinically meaningful in later life (75+), regardless of sex, whereas in midlife and early older adulthood, subjective complaints were not associated with poorer objective performance.

Across all demographic subgroups, no significant differences emerged between SCD groups and controls on the MAT and Stroop Interference task. However, men aged 75+, those with SCD + Worry performed worse than controls on the AFT. These findings are partially in line with previous studies reporting either lower (e.g., Kim et al., 2020; López‐Higes et al., 2017; Morrison & Oliver, 2022; Wolfsgruber et al., 2020) or comparable (e.g., Caillaud et al., 2020) executive functions in individuals with SCD relative to controls. Unexpectedly, men with SCD + Worry outperformed controls on the COWAT in the 55–64 and 65–74 age groups, and women in both SCD groups (+/− worry) outperformed controls in the 65–74 age range.

In the memory domain, men aged 45–54 and 55–64 with SCD‐No Worry performed better than controls on the immediate recall of the RAVLT. In contrast, in the 75+ group, both SCD groups (+/− worry) had poorer delayed recall than controls. Among women, those with SCD‐No Worry also had lower delayed recall than controls in the 75+ group. The findings in the 75+ age group align with previous work showing lower episodic memory performance in individuals with SCD (e.g., Benito‐León et al., 2010; Kim et al., 2020; Morrison & Oliver, 2022; Park et al., 2019) and SCD‐related worry (Pavel et al., 2022) compared to those with no SCD. However, they contrast with Heser et al. (2019), who reported lower delayed recall among women with SCD but not men. Although prior work suggests that women with SCD may be more perceptive of subtle cognitive changes (Heser et al., 2019; Pérès et al., 2011), experience more cognitive decline (Oliver et al., 2022) and have a higher likelihood of progressing to dementia (Heser et al., 2019) compared to men, our findings indicate that in those aged 75 and older, both men and women with SCD exhibit poorer delayed recall. This suggests that in later life, SCD may reflect emerging cognitive decline regardless of sex.

When considered alongside the executive function tasks, the findings in the memory domain showed a more consistent pattern across men and women. This aligns with evidence suggesting that episodic memory tasks may be particularly sensitive to early cognitive changes in preclinical AD (Mortamais et al., 2017), and subtle deficits in this domain are frequently observed in SCD (Zhou et al., 2025). Although prior work has reported sex differences in cognitive trajectories in individuals with SCD, our cross‐sectional findings did not reveal sex‐specific associations. Importantly, this should not be interpreted as evidence that sex stratification is unnecessary, as it remains possible that sex‐specific effects may emerge longitudinally. Analysis of continued CLSA follow‐ups can help clarify whether men and women with SCD display different cognitive trajectories over time.

It should be noted that individuals with SCD performed as well as—or in some cases better than—controls on both executive function and memory tasks in the younger age groups (i.e., 45–54, 55–64, 65–74). This pattern supports evidence suggesting that SCD in midlife is often driven by affective or psychosocial factors rather than neurodegenerative changes (Jessen, Amariglio, et al., 2014). However, because the CLSA cohort is highly educated (approximately 80% have a university degree) and generally healthy, this pattern may also reflect sample‐specific characteristics. Higher educational attainment has been associated with increased reporting of SCD (Comijs et al., 2002), possibly reflecting greater metacognitive awareness. Moreover, highly educated individuals may have higher baseline cognitive ability, making them more sensitive to subtle cognitive changes that do not correspond to measurable deficits.

Although the CLSA cognitive battery was designed for use in these middle‐aged groups (see Tuokko et al., 2017), ceiling effects may have led to reduced sensitivity in the cognitive tasks. Cognitive differences in SCD are often subtle and may not be detected by conventional measures. In line with previous studies that have used novel, more cognitively demanding tasks to assess cognition in older adults with SCD (e.g., De Simone et al., 2023), future research should incorporate more challenging tasks to better capture early changes.

Moreover, although SCD‐related worry has been proposed as a feature that may increase the likelihood that SCD reflects underlying pathology (Jessen, Amariglio, et al., 2014), our findings did not consistently demonstrate poorer cross‐sectional cognitive performance among individuals with SCD + Worry compared to those without worry. In several instances, objective differences were associated with the presence of SCD more broadly rather than being specific to those with worry. However, it remains possible that worry may be more strongly associated with longitudinal, rather than cross‐sectional, cognitive decline. Future longitudinal analyses within the CLSA will be important for clarifying whether SCD‐related worry predicts differential cognitive trajectories over time.

### Implications

Our findings highlight the importance of considering demographic variables when interpreting subjective complaints. Several implications arise from our findings. First, memory‐based assessments appear to be more sensitive to subtle cognitive changes in SCD, specifically when the questions used to assess SCD focus on memory. In both men and women aged 75+, episodic memory performance consistently differentiated those with and without SCD, whereas executive function measures were less informative and, in the case of the COWAT, produced unexpected results. As previously discussed, however, subtle cognitive deficits in SCD are not limited to the memory domain, and our findings should not be interpreted as such. Nonetheless, when time or resources are limited, clinicians and researchers may consider prioritizing memory‐based assessments for early detection efforts.

Second, our findings suggest that the clinical relevance of SCD appears to increase with age. In the younger age groups, individuals with SCD often performed similarly to or better than controls, whereas in the oldest age group (75+), those with SCD showed poorer performance across domains. This pattern indicates that SCD may become more clinically meaningful in later life in both men and women, potentially reflecting early cognitive change. This highlights older adults with SCD as a particularly important subgroup for targeted monitoring and early screening. These age‐related patterns may also inform biomarker research. As evidence increasingly links SCD to AD biomarkers, our findings suggest that individuals in older adulthood, rather than midlife, may be especially informative for fluid biomarker and neuroimaging studies seeking to identify early AD‐related changes.

Finally, our study also extends prior research not only through its findings but also through its analytic approach. Despite their clinical and theoretical relevance, sex and age are usually treated as covariates, rather than moderators, of cognitive performance in much of the existing literature. By examining SCD‐related differences in cognitive performance within each sex and age group, our analyses revealed subgroup‐specific associations that could have otherwise been masked in a covariate‐adjusted model. Our findings provide a more nuanced understanding of how subjective cognitive decline relates to objective cognitive outcomes in different groups, ultimately highlighting the importance of considering demographic characteristics when evaluating SCD in both clinical and research settings.

### Limitations and future directions

While we aimed to address key methodological limitations in prior studies, such as limited assessment of objective cognition in domains other than memory and inadequate control of confounding variables (e.g., depressive symptoms, neurological disorders), our study is not without limitations. Due to previously reported differences in cognitive performance between English and French speakers in the CLSA (Tuokko et al., 2017), only participants who completed the neuropsychological battery in English were included in the current analyses. As such, this may limit the generalizability of our findings to the general Canadian population.

Additionally, SCD was assessed via questions addressing only memory‐related concerns, which do not capture perceived changes in other cognitive domains (e.g., language, executive function). This domain‐specific assessment may partially explain why differences were more evident in memory tasks than executive function tasks. Future studies should incorporate more comprehensive assessments, such as the Everyday Cognition (ECog) Scale (Farias et al., 2008) and Cognitive Change Index (CCI; Rattanabannakit et al., 2016), to capture perceived decline across multiple domains. An additional advantage of such measures is that they provide a continuous measure of SCD, allowing for the assessment of severity of concerns (Molinuevo et al., 2017). Greater SCD severity has been associated with poorer cognitive performance (Burmester et al., 2016) and increased likelihood of cognitive decline (Amariglio et al., 2018; Dufouil et al., 2005; Zhou et al., 2025).

Lastly, this study reports only cross‐sectional analyses. Because SCD was first assessed at the first follow‐up of the CLSA, baseline SCD status was unknown. Cross‐sectional studies often yield inconsistent results regarding cognitive performance in SCD, while longitudinal studies show stronger associations between SCD and future cognitive decline (Rabin et al., 2017; Zhou et al., 2025). However, data collection in the CLSA is ongoing, thus offering an excellent opportunity to examine the relationship between SCD and cognition longitudinally.

## CONCLUSION

This study examined how the relationship between SCD status and cognition varies by age and sex within the CLSA. In the 75+ age group, SCD was associated with poorer episodic memory performance in both men and women and lower AFT performance in men. In younger age groups, subjective complaints were not consistently associated with objective cognitive differences. These findings suggest that the clinical significance of SCD may increase with age and highlight the importance of demographic context when interpreting subjective cognitive concerns. Future research should investigate cognitive differences associated with SCD and related worry longitudinally, including through continued follow‐up in the CLSA, to improve our understanding of factors shaping cognitive trajectories in this population.

## AUTHOR CONTRIBUTIONS

**Sofia Marinou:** Methodology; data curation; formal analysis; visualization; writing – original draft; writing – review and editing. **Vanessa Taler:** Conceptualization; methodology; supervision; resources; writing – review and editing.

## FUNDING INFORMATION

Funding for the Canadian Longitudinal Study on Aging (CLSA) is provided by the Government of Canada through the Canadian Institutes of Health Research (CIHR) under grant reference: LSA 94473 and the Canada Foundation for Innovation, as well as the following provinces, Newfoundland, Nova Scotia, Quebec, Ontario, Manitoba, Alberta and British Columbia. This research was supported by the Canadian Institutes of Health Research (CIHR) [Funding Reference Number: 530767].

## CONFLICT OF INTEREST STATEMENT

The authors declare no conflicts of interest.

## DISCLAIMER

The opinions expressed in this manuscript are the authors' own and do not reflect the views of the Canadian Longitudinal Study on Aging.

## Data Availability

The data that support the findings of this study are available from Canadian Longitudinal Study on Aging (https://www.clsa‐elcv.ca) for researchers who meet the criteria for access to de‐identified CLSA data.
